# 3D-printed gelatin methacrylate (GelMA)/silanated silica scaffold assisted by two-stage cooling system for hard tissue regeneration

**DOI:** 10.1093/rb/rbab001

**Published:** 2021-03-13

**Authors:** Eunjeong Choi, Dongyun Kim, Donggu Kang, Gi Hoon Yang, Bongsu Jung, MyungGu Yeo, Min-Jeong Park, SangHyun An, KyoungHo Lee, Jun Sik Kim, Jong Chul Kim, Woonhyeok Jeong, Hye Hyun Yoo, Hojun Jeon

**Affiliations:** 1 Research Institute of Additive Manufacturing and Regenerative Medicine, Baobab Healthcare Inc, 55 Hanyangdaehak-Ro, Ansan, Gyeonggi-do 15588, South Korea; 2 Department of Mechanical Engineering, Korea Polytechnic University, Sangidaehak-ro, Siheung, Gyeonggi-do 15073, South Korea; 3 Medical Device Development Center, Daegu-Gyeongbuk Medical Innovation Foundation (DGMIF), 80, Cheombok-ro, Dong-gu, Daegu 41061, South Korea; 4 Laboratory Animal Center, Daegu-Gyeongbuk Medical Innovation Foundation (DGMIF), 80 Cheombok-ro, Dong-gu, Daegu 41061, South Korea; 5 Department of Plastic and Reconstructive Surgery, Dongsan Medical Center, Keimyung University College of Medicine, 1035 Dalgubeol-daero, Dalseo-gu, Daegu 42601, South Korea; 6 Institute of Pharmaceutical Science and Technology, College of Pharmacy, Hanyang University, 55 Hanyangdaehak-Ro, Ansan, Gyeonggi-Do 15588, South Korea

**Keywords:** 3D bioprinting, gelatin methacrylate, silanated silica, cooling system, human mesenchymal stem cells

## Abstract

Among many biomaterials, gelatin methacrylate (GelMA), a photocurable protein, has been widely used in 3D bioprinting process owing to its excellent cellular responses, biocompatibility and biodegradability. However, GelMA still shows a low processability due to the severe temperature dependence of viscosity. To overcome this obstacle, we propose a two-stage temperature control system to effectively control the viscosity of GelMA. To optimize the process conditions, we evaluated the temperature of the cooling system (jacket and stage). Using the established system, three GelMA scaffolds were fabricated in which different concentrations (0, 3 and 10 wt%) of silanated silica particles were embedded. To evaluate the performances of the prepared scaffolds suitable for hard tissue regeneration, we analyzed the physical (viscoelasticity, surface roughness, compressive modulus and wettability) and biological (human mesenchymal stem cells growth, western blotting and osteogenic differentiation) properties. Consequently, the composite scaffold with greater silica contents (10 wt%) showed enhanced physical and biological performances including mechanical strength, cell initial attachment, cell proliferation and osteogenic differentiation compared with those of the controls. Our results indicate that the GelMA/silanated silica composite scaffold can be potentially used for hard tissue regeneration.

## Introduction

A scaffold is a biomaterial alternative that structurally supports the damaged areas of the internal body and serves a role as an artificial tissue and organ or a prosthesis [1, 2]. The scaffold should replace the tissue or organ directly proportional to their maturation rate [3, 4]. Therefore, the scaffold structure must possess the mechanical properties required to perform their role while being coexisted with the host tissues within the body, and the biological properties similar to that of the human body to induce tissue growth and maturation [5, 6]. In general, biodegradable polymers are used as the main ingredient to fabricate the cell carrier. Biodegradable polymers can be grouped into two categories: synthetically and naturally derived polymers. Especially, there have been an increasing number of reports that utilize protein-based hydrogels (collagen, alginate, chitosan, silk-fibroin and gelatin etc.) that have similar characteristics to native tissues to improve bio-functionality, such as cell culture, proliferation and differentiation [7–9].

Protein-based hydrogels are widely used as bioink materials for 3D bioprinting due to the suitable characteristics of protein (biodegradability, biocompatibility and cytophilicity). Recently, there are many ongoing studies on the utilization of protein-based 3D scaffolds necessary to regenerate organs, such as bone and cartilage [10–14]. For example, gelatin is a natural polymer in a single-helix form derived by denaturation of triple-helix structured collagen that constitutes the skin, bone and ligament of animals [15–17]. Gelatin is easily dissolved in water and acetic acid, and due to its excellent biocompatibility, it is widely used in the field of tissue engineering [18, 19]. Since it has similar composition as collagen, it is often used for regeneration of bones and cartilages [18–20]. However, gelatin, which has excellent biocompatibility, is not suitable for the use in bone tissue regeneration because of its thermal degradation at body temperature (37°C). To address this issue, there have been efforts in overcoming the rapid degradation of gelatin by using chemical crosslinkers such as genipin or glutaraldehyde [21, 22]. However, due to the toxicity caused by the chemical crosslinking process, the biocompatibility can be compromised.

Gelatin can be functionalized into gelatin methacrylate (GelMA) by introducing methacrylate group in which the mechanical strength can be improved through a photo-crosslinking process [23]. Therefore, it has been used not only in bone regeneration but also in regeneration of many different tissues. Previous examples of scaffold fabrication for tissue regeneration using GelMA as a bioink are summarized in Table 1 [24–31]. In a study of Byambaa *et al*. [24], a bone-like tissue structure was fabricated composed of GelMA using an extrusion-based 3D printing method. The fabricated hollow vascular structure was seeded with human umbilical vein endothelial cells (HUVECs)/mesenchymal stem cells (MSCs) and cultured for 21 days. As a result, high cell viability and a successful formation of bone and vascular structures was achieved. However, a rapid decomposition of the structure due to the low degree of methacrylation affected the mechanical structural stability and posed an issue in maintaining the bone tissue structure for 21 days [24]. In another study, nanosilicates were incorporated to enhance the physical and biological properties of GelMA bioink [27]. The addition of 2 wt% nanosilicates resulted in fourfold increase in compressive modulus compared to that of pure GelMA. Furthermore, the incorporation of the nanosilicates significantly increased the viscosity of the GelMA solution at 37°C. The shear-thinning properties of the GelMA/nanosilicate bioink enabled the printing of 3D structures. Finally, the nanosilicates enhanced cellular activities such as cell adhesion, proliferation and osteogenic differentiation. However, printing of high-resolution structures might be challenging. GelMA-based biomaterials for tissue engineering, especially applicable to bones, have shown shortcomings in mechanical properties, degradation rate, structure stability, as well as biological activities, which need improvements. Furthermore, for the clinical applications of GelMA, considerable studies on biological activities including osteo-immunology are necessary.

**Table 1.** Previous works on bioprinting using GelMA hydrogel

Printing method	Materials	Cell type	Target tissue	Mechanical properties	Ref.
Extrusion-based 3D bioprinting	GelMA	HUVECs hMSCs	Bone	5.5–7.5 kPa (Compressive modulus)	[24]
Co-extrusion 3D bioprinting	GelMA Alginate	HUVECs hi-PSCs Cardiomyocytes	Heart	4.3–26.5 kPa (Elastic modulus)	[25]
Extrusion-based 3-D bioprinting	PCL GelMA USPIO nanoparticle	BMSCs	Bile duct	17.41 kPa (Compressive modulus) 5.03 kPa (Young’s modulus)	[26]
Extrusion-based 3D bioprinting	GelMA nanosilicates	NIH MC3T3-E1	Bone	4.7–12.9 kPa	[27]
Stereolithographic 3D printing	PEGDA GelMA	Mouse NSCs	Neural tissue	340–560 kPa (Compressive modulus)	[28]
Two-step crosslinking strategy printing	GelMA Gelatin	BMSCs	Bone	4.44–5.26 kPa at 25°C 3.04–4.74 kPa at 37°C (Young’s modulus)	[29]
Inkjet printing	PEG GelMA	hMSCs	Bone cartilage	35–78 kPa (Compressive modulus)	[30]
Modified extrusion 3D bioprinting	GPGs	HUVECs	Soft tissue	1.8–7.0 kPa (Young’s modulus)	[31]

BMSCs: bone marrow stem cells; GelMA: gelatin methacrylate; GPGs: gelatin methacrylate physical gels; HDFs: human dermal fibroblasts; hi-PSCs: cardiomyocytes-induced pluripotent stem cell cardiomyocytes; hMSCs: bone marrow-derived human mesenchymal stem cells; HUVECs: human umbilical vein endothelial cells; NSCs: neural stem cells; PCL: polycaprolactone; PEG: polyethylene glycol; PEGDA: polyethylene glycol diacrylate.

Another essential material used for bone regeneration is bioceramics, such as hydroxyapatite (HA), tricalcium phosphate (TCP) and bioactive glasses (BG) [32–34]. In particular, silica is known as an effective material in the bone maturation due to its osteoinductive properties and its silanol groups actively interact with both calcium and phosphate ions [35]. However, pure silica scaffolds have low formability and high brittleness making it inapplicable for bone tissue regeneration, so it is used in conjunction with synthetic polymers, such as poly (lactic-co-glycolic acid) (PLGA), poly (l-lactic acid) (PLLA) and polycaprolactone (PCL) [36–38]. In our previous reports, we demonstrated the fabrication of composite scaffolds using surface modified silica and PCL solution [39, 40]. PCL was used to compensate the low mechanical strength of silica, while the surface of silica was functionalized by treating with vinyltrimethoxysilane (VTMS) to enhance the hydrophilicity [41, 42]. The scaffold containing surface-modified silica showed an improved tensile Young’s modulus compared to the un-modified sample by about 23% [39]. In addition, an increasing silica content resulted in an excellent water-uptake ability indicating an improved hydrophilicity as well as commendable *in vitro* results including stable initial adhesion, proliferation and differentiation of cells.

In this study, we hypothesized the synergistic effects between GelMA and silanated silica particles resulting in increased mechanical and biological properties for the use in manufacturing scaffolds targeting hard tissue regeneration. To obtain scaffolds with desired geometry, temperature controllable 3D printing system was developed in which the thermal dependent viscosity change could be minimized during the printing process. For the stabilization, the printed samples were exposed to UV light during and after the printing process. Three groups of scaffolds including GS 0 (15 wt % GelMA), GS3 (3 wt% silica with 15 wt% GelMA) and GS 10 (10 wt% silica with 15 wt% GelMA) were prepared, and their Si content, compressive modulus, surface roughness and water-absorbability were measured. In addition, human mesenchymal stem cells (HMSCs) were seeded onto the prepared scaffolds and evaluated the corresponding cellular responses such as cell proliferation, adhesion, morphology and differentiation.

## Materials and methods

### Synthesis of gelatin methacryloyl (GelMA) hydrogels

To prepare GelMA hydrogel, we referred to the synthesis method of Kuo et al. [43]. Briefly, 15 wt% of gelatin (gel strength of 175 g) and Dulbecco’s phosphate buffer (DPBS) were added to a round-bottom flask and allowed to dissolve at 50°C for 1 h. When gelatin was fully dissolved in DPBS, the same amount of methacrylic anhydride (MA) was added dropwise and allowed to react for 3 h. When the mixture became opaque after 3 h, 3–5 times of initial DPBS amount was added to terminate the reaction, followed by dividing the mixture and adding to the dialysis tubes to dialyze against water at 40°C for 1 week. The solution in the dialysis bag was then centrifuged, added with 0.5 wt% of photoinitiator (Irgacure 2959, Sigma-Aldrich) and freeze-dried at 80°C for 1 week. Until further use, it was stored at −80°C.

### Silane-modification of silica particles

To prepare the silica particles (800 nm, Sukgyung AT, South Korea) treated with silane, VTMS was used as the silane coupling agent. 2,6-di-tert-butyl-4-methylphenol, 1,2-dichlorobenzene and VTMS (Sigma-Aldrich). Prior to the silane reaction, 100 g of silica was added to 200 g of ethanol and it was heated to reflux for 3 h, followed by centrifugation to separate silica. It was then washed with ethanol twice and separated silica by centrifuge, before drying in an oven at 80°C overnight. After that, 100 g of the dried silica and 250 g of 1,2-dichlorobenzen were charged in a round-bottom flask and sonicated for 1 h. The precipitated silica, 10 g of VTMS and 2,6-di-tert-butyl-4-methylphenol at 0.1% of the solid content were vortex-mixed for 3 min and allowed to react at 60°C for 6 h. Finally, after centrifugation of the silanated silica particles, they were washed with 2-butanone.

### Rheological testing of the bioinks

To evaluate the rheological properties of the fabricated samples with various concentrations (0, 3 and 10 wt%) of silanated silica particles, complex viscosity η (Pa.s) dependent on temperature was measured. For the measurements, rotational rheometer (KINEXUS pro; Malvern Instruments, Surrey, UK, 40 mm diameter, 1 mm gap) was used. Shear rate was kept at 1 s^−1^ and a temperature sweep (ranging between 0°C and 40°C, and ramping rate = 1°C min^−1^) was conducted with a constant strain of 1% and a frequency of 1 Hz.

### Fabrication of GelMA/silanated silica composite scaffolds

To prepare the composite scaffolds, we used a 3D bioprinter that combines a cooling stage and cooling jacket made of a peltier material allowing easy control of the rheological properties of GelMA. Furthermore, UV light system was equipped to the printer for concurrent UV crosslinking of GelMA. For the preparation of the scaffold solution, 15 wt% GelMA, the main ingredient, was dissolved in PBS and blended with silanated silica particles (0, 3 and 10 wt%) using a syringe. After that, the well-blended composite materials were added to a stainless syringe and the temperature of the cooling jacket was controlled to be 5–9°C prior to 3D printing process. During the printing process, the cooling stage was set to be 3–7°C, while UV light (360–380 nm) intensity of 10 mW cm^−2^ was exposed for 3 min for curing. The process was carried out at a room temperature of 22 °C. A 25 G (300 μm) gauge nozzle was used with a nozzle feed rate of 3.0 mm s^−1^ and an air pressure of 0.95 ± 0.05 MPa. In this study, samples with 0, 3 and 10 wt% of silica contents were prepared, which are referred to as GS 0, GS 3 and GS 10, respectively.

### Swelling and degradation test

To observe the swelling ability of the prepared samples, the protocol suggested by Yoon et al. [44] was used. In between two slide glasses spaced 1 mm apart, 200 µL of each GelMA solution was pipetted and 10 mW cm^−2^ of UV light was exposed for 1 min. After the crosslinking process, each sample was placed in PBS at 37°C for 24 h. After removing PBS, the amount of swollen hydrogel was recorded. The sample was then freeze-dried, and the weight of the dried polymer was measured. The mass expansion ratio was calculated as: $\mathrm{swelling}\;\mathrm{ratio}=\frac{\left(w_{24}-w_{i}\right)}{w_{i}}$, where *w*_24_ indicates the swollen sample after 24 h and *w_i_* indicates the initial weight of the dried sample before soaking. A total of five samples were measured per each group.

Referring to a previous report [45], the prepared GelMA/silica scaffolds were soaked in Dulbecco modified Eagle medium (DMEM) and the sample mass change was observed over 21 days to simulate the extent of *in vitro* biodegradation. Before soaking the samples in the medium, they were sterilized under UV light for 1 h, and subsequently soaked in 70% ethanol for 30 min. Then, they were soaked in the medium and incubated at 37°C. At pre-determined time intervals, the medium was pipetted and the scaffold was freeze-dried for 24 h to determine the residual weight (*W*_r_) of each sample. The biodegradation characteristics of the GelMA composite scaffolds were expressed as a percentage of the residual weight (*W*_r_) relative to the mass before degradation (*W*_s_), as follows:

$$Q_{d}=\frac{W_{r}}{W_{s}}\times 100$$

### Characterization of the composite scaffolds

To observe the surface morphology of the fabricated composite scaffolds, a scanning electron microscope (SEM; NOVA NanoSEM 450, FEI Co) and optical microscope (BX FM-32; Olympus, Japan) connected to a digital camera were used. In addition, energy-dispersive spectroscopy (EDS) was conducted to confirm the uniformity of silica distribution within the fabricated scaffolds. To analyze the surface roughness depending on the silica content in the fabricated scaffolds, laser scanning microscope (LSM: VK-9710, Keyence, Japan) was used to measure the average roughness (*R*_a_) and root mean square roughness (*R*_rms_). To obtain a quantitative value of roughness, 30 points of a sample were randomly selected.

To confirm the synthesized GelMA and the VTMS silane group of surface-modified silica, we used Fourier transform infrared (FT-IR) spectrometer (model 6700; Nicolet, West Point, PA). The functional groups before and after the synthesis of GelMA as well as before and after the reaction of silica particles with VTMS were compared for analysis. Infrared spectra of the modified composite represent the average of 30 scans between 500 and 4000 cm^−1^ at a resolution of 8 cm^−1^.

To study the mechanical properties of the three types of scaffolds, a compressive modulus test was performed using Universal Testing Machine (Model 3345, Instron Co., Norwood, MA). For the measurement, the samples were cut into small strips of 1.5 × 2.0 × 2.0 mm^3^ and the compressive speed was set at 0.1 mm s^−1^ to obtain stress–strain curves. All values were expressed as the means ± SD (*n* = 5).

To observe the change in water absorption ability of the composite scaffolds depending on the silica content, one droplet of water (10 μL) mixed with a red dye was carefully deposited onto the surface of the scaffolds, and the degree of absorption was observed at different time intervals (0 and 10 s). To measure the water absorption rate, it was soaked in distilled water for 2 h and the mass before and after soaking was compared. The rate of absorbed water was calculated as (%) = (*W*_2h_ –*W*_0_)/*W*_0_ × 100, where *W*_2h_ is the weight of scaffolds after 2 h and *W*_0_ is the initial weight of the scaffolds.

### *In vitro* cell culture

For cell culture, the GelMA/silanated silica scaffolds (5 mm^3^ × 5 mm^3^ × 2.4 mm^3^) were prepared and sterilized with 70% ethanol and UV light for 3 h each, and then placed in a culture medium overnight. Human mesenchymal stem cells derived from bone marrow (hMSCs-BM; Promo Cell, Heidelberg, Germany) were cultured in the composite scaffolds to observe the cellular behavior. hMSCs were cultured in mesenchymal stem cell growth medium 2 (Promo Cell, Heidelberg, Germany). Each scaffold is seeded with cells at a density of 5 × 10^4^ and put in incubation (5% CO_2_ at 37°C), with medium exchange every second day. The cells were cultured in DMEM low glucose containing 50 μg mL^−1^ vitamin C and 10 mM β-glycerophosphate.

### Proliferation of viable cells

To confirm the cytotoxicity of the prepared composite scaffolds, we executed the live/dead cell assay. For obtaining images of live/dead cells, the composite scaffolds were exposed to 0.15 mM calcein AM and 2 mM ethidium homodimer-1 for 45 min in an incubator after 24 h of cell culture. In the captured images, green and red indicate live and dead cells, respectively.

For quantitative analysis of cell viability, the composite scaffolds seeded with hMSCs were placed in a 96-well culture plate and measured using a CellTiter-Glo^TM^ assay kit (Promega^®^, Madison, WI). Evaluation was performed at 1, 3, 7 and 14 days for each group following the protocol provided by the manufacturer. The absorbance was recorded at 750 nm using a microplate reader (EL800; Bio-Tek Instruments, Winooski, VT). For each individual experiment, six samples were used. The number of proliferated cells was normalized according to an established standard curve.

### Total protein content

The bicinchoninic acid (BCA) protein assay (Pierce Kit; Thermo Scientific) was used to determine the total protein content after 24 h, 7, 14 and 21 days of cell culture. PBS and 1 ml 0.1% Triton X-100 were used for washing and lysing process, respectively. The mixture of 200 μL BCA working reagent and 25 μL aliquot of the lysate was prepared and placed in an incubator for 30 min at 37°C. A plate reader measured the protein concentration from the absorbance at 562 nm to normalize the ALP activity and calcium deposition. After quantification, an aliquot of 2 μg of the separated protein was stored at −70°C for western blotting.

### Western blotting

For electrophoresis, sodium dodecyl sulfate-polyacrylamide gel electrophoresis (SDS-PAGE) was used at 100 V for 2 h to obtain 2 µg of proteins. After, the proteins were transferred to the polyvinylidene difluoride (PVDF) membrane. The membrane was immersed and stirred in 5% skim milk in tris-buffered saline/tween20 (TBST) for blocking. Then, the membrane was incubated with the primary antibodies and stirred for 12 h at 60 rpm at 4°C. To remove the unconjugated antibodies, the membrane was washed three times with TBST. The membrane was further incubated with the secondary antibodies diluted 1:3000 with 5% skim milk in TBST at room temperature. The protein bands were detected with chemiluminescent (Cat# 34577, Thermo scientific, Rochford, IL). In order to quantitatively analyze the bands, the density was quantified using ImageJ software (National Institutes of Health, Bethesda, MD) and plotted as a graph.

### Dapi/phalloidin/wheat germ agglutin (WGA) and osteopontin staining

For the observation of the initial cell morphology of hMSCs seeded in composite scaffolds, the cells were fixed in 4% paraformaldehyde at room temperature. After washing in PBS, to block unspecific protein interactions, the samples were put in blocking solution (0.3% Triton X-100, 1% BSA, 10% donkey serum). Then, the samples were incubated with WGA-Alexa Fluor conjugate (Life Technologies) at 37°C for 1 h. Also, to observe the expression of osteopontin, one of the bone differentiation proteins, 10 μg mL^−1^ osteopontin primary antibody (Abcam) was incubated overnight at 4°C. After washing, FITC-conjugated secondary antibody was incubated for 1 h at room temperature. Then, the samples were washed for nuclear labeling in the cells using mounting media (within DAPI, VECTOR) and observed using an LSM 5 Exciter confocal microscope (Carl Zeiss Microscopy GmbH, Olympus, Japan).

### Alp activity and mineralization

After 7, 14 and 21 days of cell culture, the cells were fixed with 4% paraformaldehyde at room temperature and stained for alkaline phosphatase (ALP) with the TRACP & ALP double-staining kit (Takara, MK300). Briefly, immobilized samples were incubated in 250 µL of substrate solution at 37°C per scaffold to detect alkaline phosphatase. After, the reaction was terminated by washing with deionized water. Also, the ALP activity of the cells was quantified during the same amount of time using an ALP kit (procedure no. ALP-10; Sigma-Aldrich). The samples were washed with PBS and placed in 0.1% Triton X-100 contained with Tris buffer (10 mM, pH 7.5) for 10 min. 100 μL of the lysate together with 100 μL of p-NPP solution were added to 96-well tissue culture plates. The ALP activity was quantified using a microplate reader (EL800; Bio-Tek Instruments, Winooski, VT).

For the observation of calcium deposition of the cells, alizarin red S staining was performed. The cells were fixed in cold ethanol (4°C) and stained with 40 mM alizarin red S (pH 4.2) for 1 h. After washing with purified water, the specimens were incubated with 10% cetylpyridinium chloride in 10 mM sodium phosphate buffer (pH 7.0) for 15 min. For qualitative and quantitative analysis, an optical microscope was used and the optical density (OD = 540 nm) was measured using a microplate reader. All OD values were normalized to the total protein content. All values are expressed as means ± SD (*n* = 6).

### Cell-printing process

A GelMA solution was prepared, as described above. A cell suspension composed of 1 × 10^6^ hMSCs mL^−1^ in media was mixed with the prepared GelMA solution. Then, 5 mg mL^−1^ of photoinitiator (2-hydroxy-4′-(2-hydroxyethoxy)-2-methylpropiophenone (Irgacure 2959; Sigma-Aldrich)) in PBS was added to the mixture.

### Statistical analyses

The data acquired in this study are represented as means ± SD. SPSS software 10.0 (SPSS, Inc., Chicago, IL) was used for statistical analysis, and single-factor analysis of variance was used. If ∗*P* < 0.05, it was considered to indicate statistical significance.

## Results and discussion

### Synthesis of GelMA/silica composites

**Figure 1.**
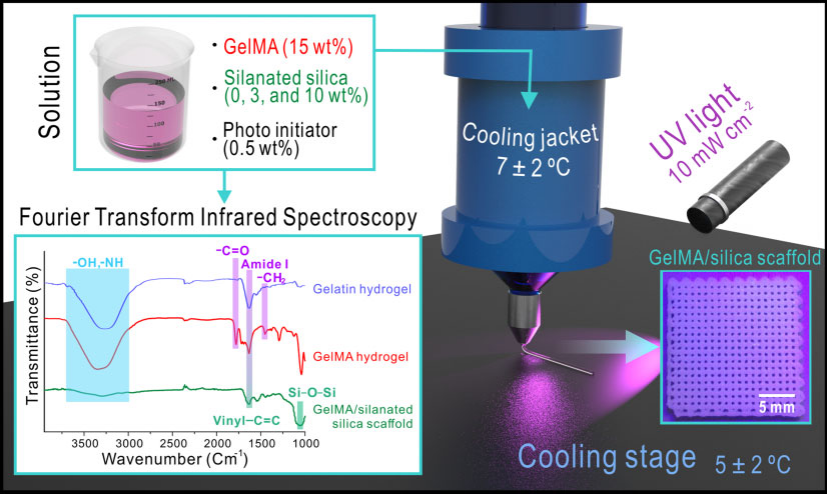
Schematically illustrated image showing the fabrication process of silanated silica-containing GelMA scaffolds.

In this study, we fabricated GelMA/silica composite scaffolds using surface-modified silica particles to evaluate their ability for physical properties as a filler for bone regeneration. Figure 1 is a schematic diagram of the manufacturing process of GelMA/silica composites. Referring to previously published methods [23, 46, 47], MA was added to functionalize the gelatin solution for the preparation of photo-crosslinkable hydrogel. After that, the gelatin-MA mixture was dialyzed and centrifuged to afford the final GelMA hydrogel. To confirm the successful synthesis of GelMA, main peaks of GelMA were found through FT-IR spectroscopy. As seen in Fig. 1, four main peaks—a broad band –OH and –NH in the 3050–3750 cm^−1^ range, –C = O at 1770 cm^−1^, amide I at 1635 cm^−1^ and –CH_2_ at 1470 cm^−1—^GelMA represents were found in the synthesized hydrogel. From these results, an effective modification from gelatin to GelMA was confirmed. Besides GelMA, silica was also added as a composite material in this study known as a potential biomaterial that can effectively regenerate bone tissues. Especially bone induction and bone formation are induced due to the silanol groups present on its surface activating the interaction with calcium and phosphate ions [48]. However, the biggest drawbacks arise from the low mechanical strength and fracture toughness when pure silica is used as a biomaterial for bone regeneration [48].

Silane coupling agents can help improve the mechanical strength and compatibility of the polymer matrix and inorganic filler interaction when inorganic particles are used as polymer fillers. [49]. The –OCH_3_ group of VTMS can react with –OH on silica surface through a hydrolysis reaction, which forms Si–CH CH_2_ groups on the silica surface. The FT-IR spectrum of prepared GelMA/silanated silica composite scaffold is shown in Fig. 1. In general, for surface-treated ceramic using VTMS, peaks corresponding to Si–O–Si antisymmetric stretching vibrations at 1089 cm^−1^ and Si–O–Si bending vibrations at 802 cm^−1^ are found. In addition, previous studies confirmed that peaks corresponding to stretching vibrations of vinyl C = C are found in a range of 1600–1680 cm^−1^ and the peaks corresponding to C–H (=CH, =CH_2_) at 2891 cm^−1^ and 2985 cm^−1^ [39, 40]. From the IR spectrum, we confirmed that VTMS-treated silica were effectively introduced to the GelMA hydrogel from the broad band peak in the 3050–3750 cm^−1^ range, Si–O–Si antisymmetric stretching vibrations at 1089 cm^−1^ from VTMS-treated silica, stretching vibrations of vinyl C = C in a range of 1600–1680 cm^−1^ from the treated silica-containing composite scaffolds.

### Rheological, swelling and degradation characteristics of GelMA/silica bioink

**Figure 2.**
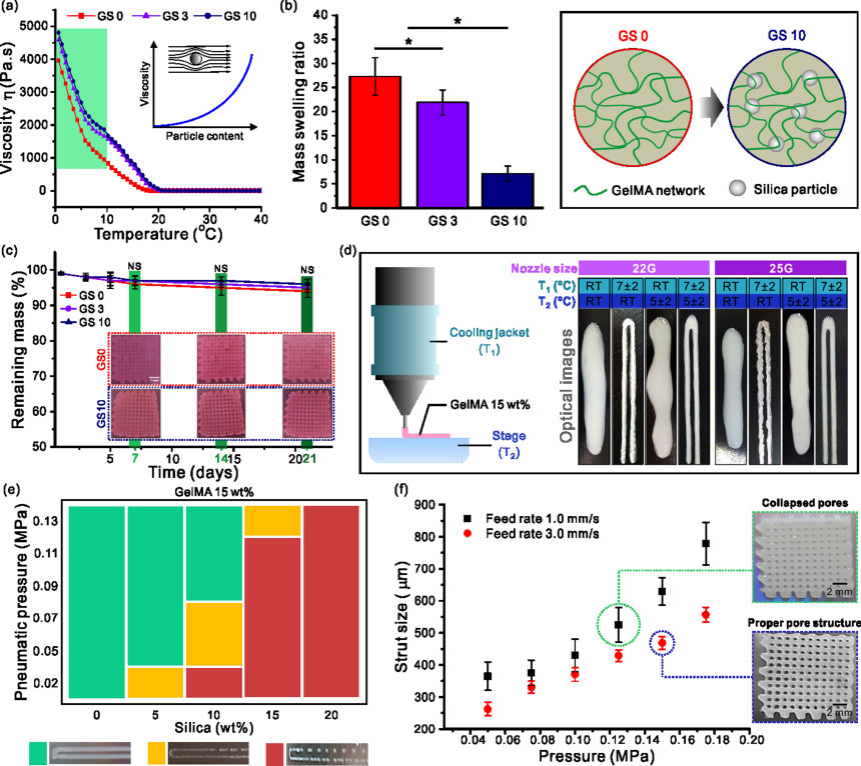
(**a**) Rheological, (**b**) swelling and (**c**) degradation test results of the scaffolds. Printability depending on (**d**) temperature of the jacket and stage and (**e**) applied pressure and silica content. (**f**) Strut size depending on the feed rate and applied pressure.

Gelatin is a temperature-sensitive polymer and its viscosity can change rapidly with temperature. For this reason, we observed the rheological characteristics dependent on silica concentrations of the prepared GelMA ink and the temperature. The graph shown in Fig. 2a indicates that the viscosity of all bioinks rapidly decreased with increasing temperature, where a clearly low value was obtained at > 20°C. Additionally, the bioink with high silica content (10 wt%) showed greater overall viscosity between 0 and 10°C compared to the other two bioinks. This phenomenon can be explained using the Krieger–Dougherty equation, $\frac{\eta }{\eta_{0}}={\left(1-\frac{\varphi }{\varphi_{m}}\right)}^{-[\eta ]\theta_{m}}$, in which η indicates the viscosity of the suspension, η_0_ indicates the viscosity of the medium, ϕ indicates the volume fraction of the particles, ϕ_m_ indicates the maximum volume fraction in the suspension and [η] indicates the intrinsic viscosity (2.5 for spheres) [50]. Thus, increased particle content results in increased viscosity. Particles in the solution hinder the flow of the liquid increasing the flow resistance which is the viscosity as illustrated in Fig. 2a. Therefore, we managed to maintain the temperature below 10°C for the printing process.

Figure 2b shows the swelling characteristics of GelMA–silica hydrogel with respect to the silica content. As shown in the graph, a distinct difference in swelling behaviors were observed between each group according to the silica content. As the silica content increased from 0 to 10%, the swelling ratio decreased from 27.3 ± 3.9–7.1 ± 1.6%. These results indicate that the silica particles reduced the swelling ability of the structures. This was due to the GelMA–silica bonding which decreased the sites for the water molecules to bond. Therefore, increased silica content caused decreased swelling ability of the GelMA scaffolds. Also, the swelling ability of hydrogels can be decreased owing to the reduced internal pore size of the hydrogel system [51]. Extensive swelling behavior may lead to decreased mechanical properties and additional compressive stress to the host tissue [52].

Furthermore, the degradation rate of scaffolds must be inversely proportional to the growth rate of the tissue [3]. Particularly, in bone regeneration, the physical properties of the scaffold material play an essential role since it is closely related to the subsequent remodeling and improving the functional recovery [53, 54]. We conducted a degradation test to investigate the effect of silica content on the degradation rate. Figure 2c shows the degradation results analyzed by soaking the prepared GelMA/silica scaffolds in DMEM media up to 21 days. As shown in the optical images and graph, the degradation rate of the scaffolds was not dependent on the silica content. Although the degradation mass showed a decreasing trend as the silica content increased, the difference compared to the sample containing no silica was not significant. This observation can be explained by the stability of GelMA being controlled to some extent through the UV curing process in turn the media was not able to penetrate into the rigid scaffold easily.

### Printability of GelMA/silica solution

A scaffold is a tissue engineered implant onto which cells must adhere and proliferate, and it must also promote angiogenesis, receive appropriate nutrients and achieve uniform differentiation for a successful tissue regeneration [2]. For this, the scaffold must have a proper porous structure (proper pore size, porosity and pore interconnectivity) based on an excellent printability [55]. However, as mentioned above, GelMA hydrogels have a very poor printability when printed at room temperature due to its rapid viscosity change with the temperature. A poor printability interferes with the accurate design of the scaffold and may play an important role in jeopardizing its physical properties by changing the shape of the scaffold.

Prior to the scaffold fabrication of GelMA/silica composite scaffolds, the printability of GelMA hydrogel was first studied. 15 wt% of GelMA hydrogel was used and the cooling system temperature was varied to optimize the printability. A nozzle gauge of 25 G was used, feed rate was fixed at 3.0 mm s^−1^ and the pressure range was 0.95 ± 0.05 MPa. Figure 2d shows the effect of the cooling jacket and stage temperature on the printed structure as well as the optical images of GelMA hydrogel print. As shown in the images, properly shaped struts were obtained only when both the jacket and stage were temperature-controlled. When only the temperature of the cooling stage was controlled, unstable struts were generated. Therefore, the samples were prepared by controlling the temperature of the cooling jacket from 5°C to 9°C and the cooling stage from 3°C to 7°C depending on the room temperature. To maintain the designed shape at the time of the sample fabrication, it was exposed to UV light during the printing process.

After optimizing the printability parameters of GelMA hydrogel, we studied the printability of the GelMA/silica solution. Figure 2e shows a process condition graph for the GelMA/silica composite scaffold fabrication, indicating the correlation between silica wt% and the applied pressure. For this test, the temperature (jacket = 7 ± 2°C and stage = 5 ± 2°C) and feed rate (3.0 mm s^−1^) were fixed. As seen in the graph, greater pressure was required as the silica content increased. For the fabrication of scaffolds with silica content < 10 wt%, a pressure above 0.085 MPa led to a stable fabrication. However, when the silica content increased to be > 20 wt%, unstable printing occurred in all pressure ranges. In addition, the thermal conditions were kept constant and initiated printing while varying the pressure to establish a proper printer feed rate. Figure 2f shows a graph of the strut diameter as a function of pressure when the feed rates were 1.0 or 3.0 mm s^−1^. We found that the initial strut setting value of 500 μm was achieved when 0.075 MPa was applied at a feed rate of 1.0 mm s^−1^ and when 0.095 MPa was applied at a feed rate of 3.0 mm s ^− 1^. However, at a speed of 1.0 mm s^−1^, a spreading phenomenon of the GelMA ink was observed, resulting in uneven formation of pores as seen in the optical images. Thus, we determined the feed rate and the pressure to be 3.0 mm s^−1^ and 0.095 MPa, respectively. Finally, using the optimized process parameters (Nozzle size = 25 G; cooling jacket temperature = 7°C; stage temperature = 5°C; feeding rate = 3.0 mm s^−1^; pressure = 0.095 MPa), GelMA/silica scaffolds (GS 0, GS 3 and GS 10) were fabricated where G indicates GelMA 15 wt%, S indicates the silanated silica, and the numbers indicate the silica wt%. The introduced cooling system (cooling jacket and stage made of Peltier material) enabled the precise control of the temperature for a successful fabrication.

To confirm whether the fabricated scaffolds had similar values to the initial design, we measured the strut size, pore size and porosity. For the porosity measurement, the following equation was used [56].

$$\mathrm{porosity}\;\left(\%\right)\;\mathrm{of}\;\mathrm{composite}\;\mathrm{scaffold}=1-\frac{\mathrm{apparent}\;\mathrm{density}\;\mathrm{of}\;\mathrm{scaffold}}{\mathrm{bulk}\;\mathrm{density}\;\mathrm{of}\;\mathrm{scaffold}}$$

and

$$\mathrm{apparent}\;\mathrm{density}\;\mathrm{of}\;\mathrm{scaffold}\;=\frac{\mathrm{mass}\;\mathrm{of}\;\mathrm{scaffold}}{\mathrm{volume}\;\mathrm{of}\;\mathrm{rectangular}\;\mathrm{shaped}\;\mathrm{scaffold}}$$

The strut size, pore size and porosity measurements of the scaffold are shown in Table 2. When GelMA hydrogel is printed, the strut size was slightly higher than the initially set value due to the spreading phenomenon, where the pore size was relatively smaller than the initially set value. The porosity value was generally in good agreement with the set value prior to the fabrication, and the difference between groups was negligible.

**Table 2.** Geometry of the fabricated scaffolds

	GS 0	GS 3	GS 10
Strut size (µm)	565.8 ± 32.9	558.0 ± 23.9	547.4 ± 11.8
Pore size (µm)	441.9 ± 29.4	464.6 ± 24.2	471.4 ± 19.7
Porosity (%)	71.9 ± 2.4	72.8 ± 3.7	72.5 ± 4.9

### Morphology and silica distribution of the composite scaffolds

**Figure 3.**
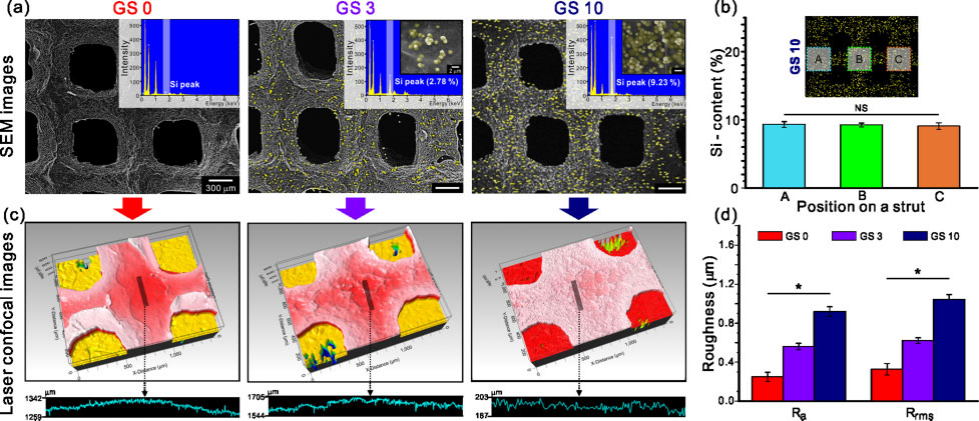
(**a**) SEM-EDS results of the scaffolds. (**b**) Dispersity of the silica particles in the GS 10 scaffold. (**c**) Laser confocal images of the scaffolds with the corresponding (**d**) roughness results.

To observe the surface characteristics including the silica content and distribution of the silica particles in the fabricated scaffolds, we obtained SEM images of the Si-mapped composite scaffold surface using EDS, as shown in Fig. 3a. Relatively uniform pore and strut size were observed in all scaffolds. Yellow color indicates the silica particles in the GelMA-based scaffolds. The EDS spectra are also shown in Fig. 3a confirming that both Si-mapping observation and values from the Si peak were representative of the silica content. Furthermore, we measured the Si content by dividing the mapping image of GS 10 into three different areas (A, B and C) to study the dispersity of silica particles. As seen in Fig. 3b, no significant difference was observed in the Si content in the different areas. Through this, we confirmed a uniform distribution of silica particles within the silica-containing scaffolds.

The silica particles embedded in the GelMA scaffolds affected the surface topology. The surface topological cue is one of the important factors in scaffold fabrication for tissue regeneration, and it has been reported that the nano-scaled roughness on the scaffold surface has a positive impact on the bone cell differentiation and gene expression [57]. In our previous report, we obtained not only a high proliferation of MSCs but also high ALP activity, osteocalcin and osteopontin expression when the optimized average roughness (*R*_a_) was approximately 600 nm in the PCL/silica scaffold [39]. To analyze the surface roughness of the fabricated scaffolds, surface profiling was performed on the center of the prepared scaffolds. In the surface profiling results as shown in Fig. 3c, it was found that a rougher profiling results were obtained with increasing silica content contained within the scaffold. From the *R*_a_ and *R*_rms_ values shown in Fig. 3d based on the corresponding surface profiling results, we confirmed that the values steadily increased according to the silica content (*R*_a_ range: 205–986 nm and *R*_rms_ range: 244–1131 nm). As a result, enhanced surface roughness was achieved in the GS 10 scaffold compared to GS 0 and GS 3 scaffolds.

### Tensile properties and water uptake ability

**Figure 4.**
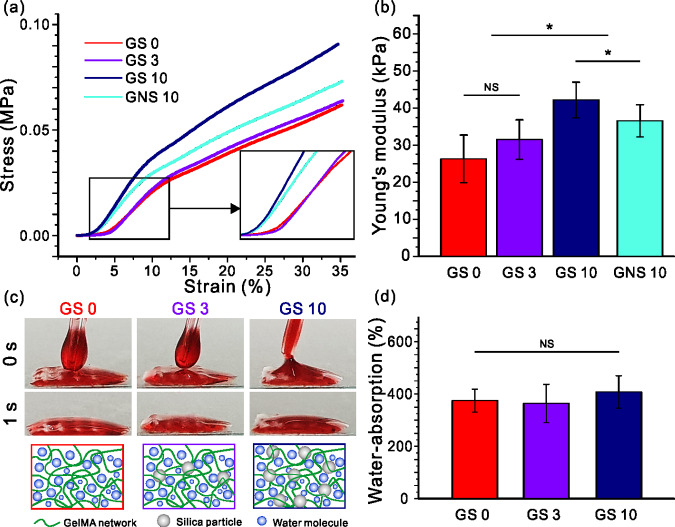
(**a**) Stress–strain curves and (**b**) Young’s modulus results of the scaffolds. (**c**) Wettability and (**d**) water absorption ability results of the scaffolds.

In general, the mechanical properties of bio-implantable scaffolds are closely associated with the structural and sustainable support functions affecting the morphology, motility and differentiation of cells [58, 59]. For example, the elastic modulus of a scaffold is correlated with the actin–cytoskeleton activity [60] and is also correlated with the extracellular signal-regulated kinase (ERK) activity and the signaling pathway of mitogen-activated protein kinase (MAPK), which can influence the osteogenic differentiation [61]. The surface of the silica particles was treated with VTMS silane coupling agent and subsequently blended with GelMA to fabricate the physically upgraded scaffolds. To evaluate the mechanical properties with respect to the silica content, a compression test was performed on the prepared scaffolds. Figure 4a and b each show stress–strain curves and Young’s modulus of the fabricated GelMA/silica scaffolds, respectively. Young’s modulus results showed that GS 10, a scaffold including 10 wt% of surface-treated silica, was elongated up to 12% compared to GS 3, and up to 23% compared to GS 0. Moreover, the GS 10 scaffold showed 10% enhanced modulus compared to the GNS 10 scaffold which is the GelMA scaffold with untreated silica particles. The modified silica particles exhibit strong repulsive forces which prevents aggregation between the particles and presents good dispersibility [62]. This effect improves the mechanical strength of the fabricated structure due to the effective dispersion of the silica particles [63]. Based on these results, we confirmed that surface-treated silica nanoparticles led to an improved mechanical strength of GelMA based scaffolds.

Besides mechanical properties, hydrophilicity and water uptake ability are crucial factors affecting the cellular behaviors. As shown in Fig. 4c, 10 μl droplet of water mixed with red dye was dropped onto the prepared samples to observe the wettability. Regardless of the silica content, we observed a rapid absorption of the droplet into the scaffolds. This phenomenon was due to the hydrophilic properties of gelatin, which is the main ingredient of the scaffold. Since the effect of silanol groups of silica was negligible, the silica content did not contribute to the observed hydrophilic behavior as schematically illustrated. This was also true for the water uptake ability which is the basic element that a scaffold should possess. When proper absorption is achieved, it can be beneficial for nutrient exchange and metabolites, helping not only cell proliferation but also metabolic functions. As seen in Fig. 4d, the absorption ability of GS 10 was not significantly different from GS 0 and GS 3. We can suggest that the silica content did not hinder the water absorption ability of GelMA.

### *In vitro* cellular activities of hMSCs on the composite scaffolds

It is known that cell division, migration and proliferation occur better in an environment with strongly adhered cells than weakly adhered cells [64]. If cells fail to adhere in the initial stage, cell proliferation and tissue division are affected, leading to cell death. Therefore, the initial cell adhesion rate is an important index to determine and confirm how effective fabricated scaffolds are in providing initial adhesion and proliferation.

**Figure 5.**
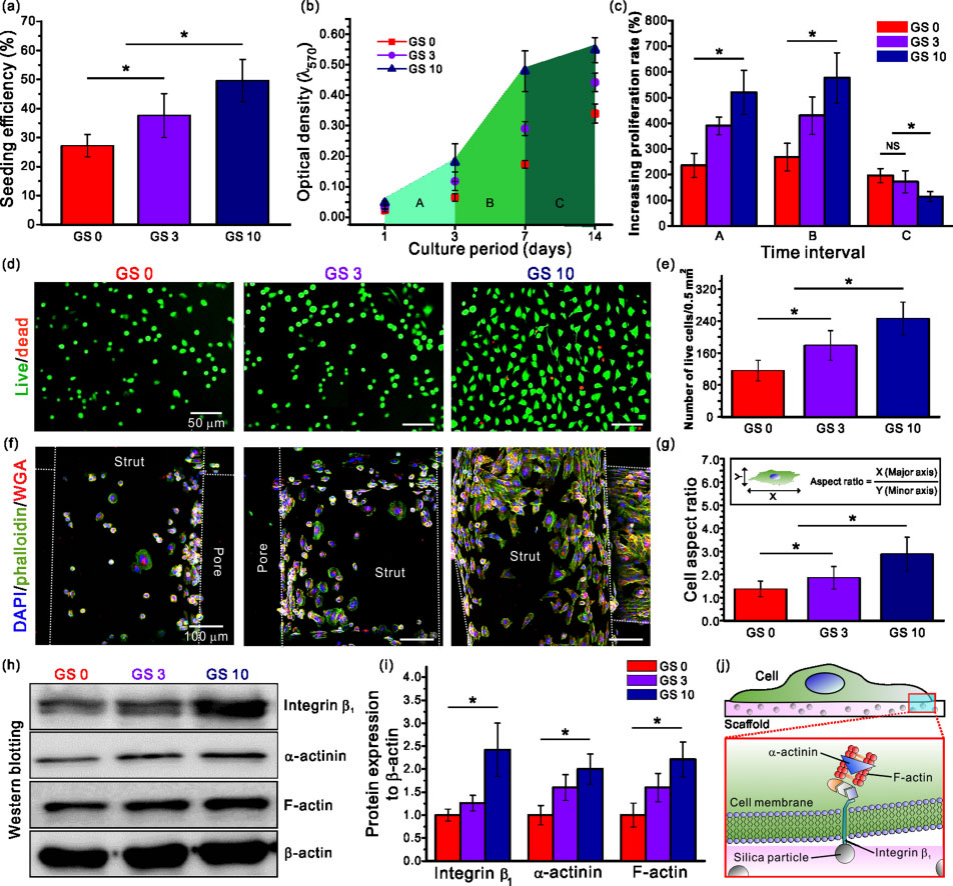
(**a**) Cell seeding efficiency of the hMSCs on the scaffolds after 4 h. (**b**) Cell proliferation results for 1, 3, 7 and 14 days and (**c**) the cell proliferation rate at different time intervals (A, B and C). (**d**) Live/dead images of the scaffolds after 24 h and the corresponding (**e**) results of the viable cell number. (**f**) DAPI/phalloidin/WGA images after 24 h and (**g**) the cell aspect ratio results. (**h**, **i**) Relative protein expression of integrin β_1_, α-actinin and F-actin. (**j**) Schematic showing the mechanism of signaling pathway.

The initial cell seeding efficiency and the proliferation rate of the bone marrow-derived hMSCs on the composite scaffolds containing silica were observed. From Fig. 5a, we confirmed that more than 51% of cells initially adhered on GS 10 scaffolds, while fewer adhering cells were observed on GS 0 and GS 3 scaffolds. This was attributed to the hydrophilic surface characteristics of the silanol group (Si-OH^−^) in silica which can lead to a good initial cell adhesion by easily bonding with the adhesion molecules of cell membranes [65]. Also, the increased roughness on the surface of the GS 10 scaffold due to the silica content positively affected the adhesion of the cells. Besides cell adhesion, an active trend in the proliferation was observed as the silica content increased as seen in Fig. 5b. The GS 10 group showed a prominent increase in the proliferation rate after day 1 compared to the other groups. However, after day 7, the proliferation started to decrease in the GS 10 group, which may be due to the fact that stem cells generally stop proliferating at a certain level and show a tendency to diversify into other tissue lineages [66]. As such, we closely observed the proliferation rate by dividing the cell culturing period into three sections (A, B and C). As shown in Fig. 5c, all groups that displayed a rapid increase in the proliferation rate in both A and B sections, showed a decreased proliferation rate in section C. However, the decreased rate was the greatest in the GS 10 scaffold (4.6-fold) due to the rapid proliferation in the early stages resulted in stem cells to stop proliferating further and start differentiating prematurely.

To confirm the initial cell adhesion and survival rate, we incubated each scaffold including hMSCs for 24 h and performed a comparative analysis of live (green)/dead (red) cells using a fluorescent microscope. As a result, the number of viable cells per area was quantitated and plotted in Fig. 5d and e, respectively. As shown in the fluorescence images, significantly higher number of cells attached and were alive in the groups containing silica compared to the pure GelMA group, which was used as a control (100%). When comparing the silica-containing groups, the number of alive cells per 0.5 mm^2^ area in GS 10 group was 2.12- and 1.37-fold greater than those in the GS 0 and GS 3 groups, respectively. These results were achieved due to the increased initial cell adhesion of the hMSCs on the GS 10 scaffolds compared to those of the other scaffolds.

Next, cellular morphology were analyzed by DAPI (blue)/phalloidin (green)/WGA (red) staining to observe initial cell morphology in detail. As shown in Fig. 5f, cytoskeleton is not properly formed in the absence of silica, evidenced by the circular-shaped cell morphology. On the other hand, in silica-containing groups, proper cytoskeleton and F-actin formation was observed due to active cell spreading and active network formation among neighboring cells. For the quantitative analysis of the cell morphology, the aspect ratio value (major axis/minor axis) was calculated based on the fluorescence images in Fig. 5g. Consequently, from the quantitative results, the greatest cell aspect ratio (2.89) was observed in the GS 10 samples indicating that the cells have experienced a cytoskeletal remodeling. The morphology of the hMSCs changed from a circular to spindle-like shape. In general, changes in cell shape are closely related to stem cell fate. According to McBeath et al. [67], high spreading of the MSCs may direct the cells to undergo osteogenic differentiation, while circular-shaped cells tend to promote adipogenesis. Thus, we can suggest that the GS10 scaffold may provide a suitable environment for the hMSCs to differentiate osteogenically.

### Initial attachment mechanisms of hMSCs on the composite scaffolds

For further studies on initial cell attachment, western blotting was performed using the collected proteins from the 24 h-cultured hMSCs (Fig. 5h and i). It is well known that the actin cytoskeleton and integrins play an important role in cell adhesion [68, 69]. Among the integrin family, integrin β_1_ is one of the adhesive proteins involved in the initial cell adhesion [70]. The β-subunit integrins are linked to F-actin through the actin-binding proteins such as α-actinin [71, 72]. Taking this into account, the levels of integrin β_1_, α-actinin and F-actin were measured. The western blotting results suggested that all expression levels were strongly dependent on the silica content in the scaffolds. The levels were increased when the concentration of silica was increased, since the targeted proteins are correlated to each other. The expression levels of the proteins were the greatest in the GS 10 scaffold containing 10 wt% of silanated silica. These results are in agreement with the initial cell seeding efficiency in which GS 10 scaffold showed significantly greater percentage compared to the other scaffolds. This may be due to the increased SiOH^−^ concentration enhancing the expression of adhesive protein integrin β_1_. These results demonstrated that integrin β_1_ played an important role as a chemical sensor at the time of initial cell adhesion after cells are seeded on the composite scaffolds. In addition, as shown in the quantitative graph in Fig. 5i, significant expression differences of α-actinin and F-actin among groups are observed. The expression was the greatest is the GS 10 group in which the cells recognized the silanol groups using integrin β_1_ influencing the adhesion mechanism. This mechanism is schematically illustrated in Fig. 5j. A signaling pathway is activated during the initial stage of the interaction between silica and hMSCs. Initially, integrin β_1_ senses the silica particle and subsequently α-actinin and F-actin are activated by integrin β_1_ which have positive impacts on cell adhesion and motility during the growth process [73–75].

### Osteogenic differentiation of hMSCs on the composite scaffolds

**Figure 6.**
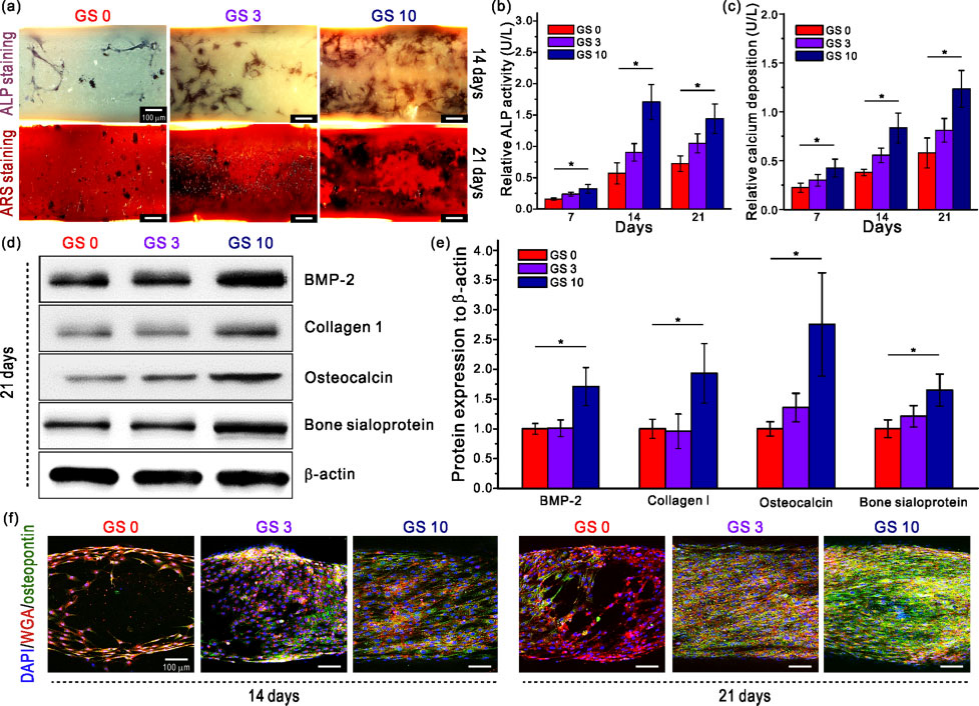
(**a**) ALP- and ARS-stained images of the scaffolds. Relative (**b**) ALP activity and (**c**) calcium deposition results. (**d**, **e**) Relative protein expression of BMP-2, collagen 1, osteocalcin and bone sialoprotein. (f) Fluorescence images of DAPI/WGA/osteopontin staining of the scaffolds.

It has been reported that silica induces bone-related gene expression such as osteopontin, osteocalcin, ALP, bone sialoprotein and bone morphogenetic protein-2 [76, 77]. To determine the effect of the prepared GelMA/silanated silica scaffold on the osteogenic differentiation of hMSCs, we performed ALP and Alizarin Red-S staining after 7, 14 and 21 days of cell culture. Initially, the differentiation process begins with cell proliferation to increase the cell density, followed by alkaline phosphatase (ALP), an early differentiation marker, production. Finally, the process culminates with calcium mineralization [78, 79]. Figure 6a shows the ALP and ARS-stained images of the hSMCs cultured on the scaffolds for 14 and 21 days, respectively. From the ALP staining optical images, dark blue section expanded and became darker with increasing silica content. Alizarin Red-S staining images also showed that sections stained in dark red was more prominent in the GS 10 group. To ensure these results, the quantitative results of ALP activity and calcium deposition are evaluated and shown in Fig. 6b and c. Figure 6b shows that the GS 10 scaffold showed significantly greater ALP activity throughout 21 days of cell culture compared to GS 0 and GS 3 scaffolds. However, the cells on GS 10 displayed a slight decrease on day 21 compared to day 14. In fact, ALP is known as an essential biochemical marker, which is highly activated in the early stages of bone formation [80]. The decrease of ALP activities after day 14 may have been due to the osteogenic marker, which is highly activated in early and middle bone formation. Moreover, the highest calcium deposition was observed in the GS 10 group in the results of calcium deposition in Fig. 6c. There were significant differences among all the groups throughout the 3-week period. As demonstrated above, the osteogenic differentiation began one week after seeding, and it became more active at weeks 2 and 3. The relative calcium deposition of the GS 10 groups was 2.90- and 1.48-fold greater than those of the GS 0 and GS 3 groups. These results indicate that the differentiation of the hMSCs was strongly influenced by the silica contained in the scaffolds.

Thereafter, in order to observe the protein expression level of osteogenic biomarkers such as bone morphogenetic protein-2 (BMP-2), collagen type 1 (COL1), osteocalcin and bone sialoprotein during the hMSCs differentiation process in the composite scaffolds, western blotting was performed. The BMP is known to have an excellent bone induction ability among osteogenic biomarkers and 15 different types have been identified thus far, where BMP-2, -4 and -7 have been reported to show strong bond induction [81]. The quantitative graph is shown in Fig. 6e based on the real-time PCR results from Fig. 6d suggests that the expression level of BMP-2 of the GS 10 group on day 21 is approximately 1.7-fold greater than that of the GS 0 group. However, differences in the BMP-2 expression level between the GS 0 and GS 3 groups were not significant regardless of the 3 wt% silica contained in the GS 3 scaffold. Therefore, we can assume that 3 wt% of silica content was not enough to induce BMP-2 expression. Another important protein is COL1, responsible for matrix deposition during biomineralization [82]. The expression of COL1 was the highest in the GS 10 group 21 days after the cell seeding, although a clear difference was not observed between the pure GelMA and the GS 3 group. Type 1 collagen is actively expressed and released in bone-forming osteoblasts, but it decreases in osteocytes. Therefore, it can be concluded that hMSCs of the GS 10 group on day 21 were in a mature stage compared to other groups. Osteocalcin is an osteogenic biomarker, which is a key factor to determine the bone formation, and a bone protein that plays the most important role in bone resorption and bone regeneration [83]. From results shown in Fig. 6e, it was found that the mRNA expression increased up to 2.8-fold on day 21 in the GS 10 group, indicating that silica has a positive effect in osteocalcin expression. Bone sialoprotein is a type of non-collagenous protein found in mineralized tissues such as bone representing about 12% of the bone protein and is highly expressed during the initial formation of bone and cementum [84]. As shown in the real-time PCR and the graph, the highest level was observed in the GS 10 group and the difference between groups proved that silica is an important influencer.

After, we stained osteopontin as an indicator of bone differentiation and maturation and used fluorescence images for qualitative observation in addition to the protein quantitative analysis of aforementioned osteogenic biomarkers. The role of osteopontin protein within the mineralized tissues involves regulating the migration, adhesion and differentiation of osteoblasts and osteocytes, inducing mineralization and regulating the growth of crystals [85]. Figure 6f shows the fluorescence images of DAPI (blue), WGA (red) and osteopontin (green) staining after 14 and 21 days of cell culture period. On day 14, cells completely covered the surface of the struts and osteopontin was visible in the GS 3 and GS 20 groups. On day 21, osteopontin was observed in all groups. However, differences in the expression level according to the silica content became more significant on day 21, indicating that silica is responsible for increasing the expression of osteopontin, which subsequently promotes the mineralization of osteocytes. After 21 days, the surface coverage of osteopontin in the GS 10 scaffold was greater than 65%.

### Cell-encapsulated composite scaffolds

**Figure 7.**
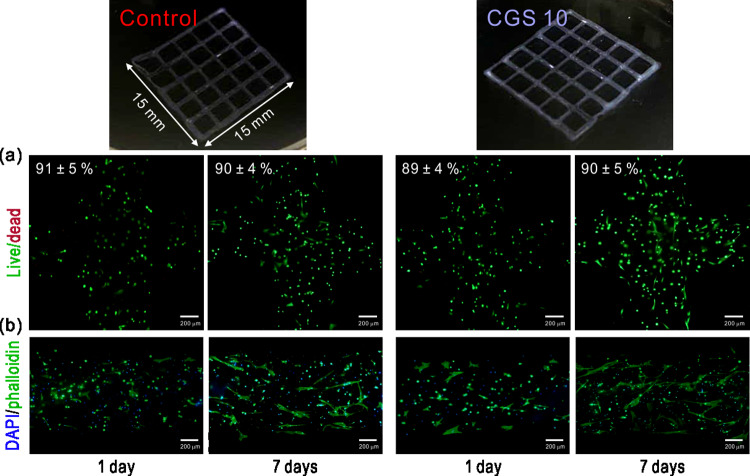
(**a**) Live/dead and (**b**) DAPI/phalloidin stained images of the cell-printed scaffolds.

Finally, we attempted to fabricate a cell-laden GS 10 (CGS 10) scaffold by employing a 3D cell-printing system. Figure 7 shows the optical and fluorescence images of live/dead and DAPI/phalloidin-stained samples after 1 and 7 days. As a result, high cell viability was achieved throughout the culture periods in both the control (cell-laden GelMA) and CGS 10 scaffolds (Fig. 7a). As seen in the fluorescence images in Fig. 7b, the hMSCs in the CGS 10 scaffold showed a more stretched morphology compared to those in the control group. This indicates that the incorporated silica particles positively influenced the hMSCs embedded in the GelMA. Although further study is needed, these results show the possible applicability and efficacy of the silica particles in cell-printing systems and on the hMSCs, respectively.

In summary, we have examined the synergetic effects of GelMA and silica to promote osteogenic differentiation of hMSCs. As described above, integrin β_1_ of hMSC significantly affected the initial cell survival by interacting with SiOH^-^ in the initial adhesion stage. Also, the cells showed high activities during the growth process due to an increased expression of α-actinin and F-actin activated by integrin β_1_. The initial activities of cells resulted in differences in proliferation, causing increased expression of proteins such as BMP-2, COL1, osteocalcin and bone sialoprotein, which in turn led to significant differences in osteogenesis.

## Conclusions

In this study, 3D-printed GelMA-based scaffolds combined with different concentrations of silanated silica (0, 3 and 10 wt%) were developed using a two-stage cooling system and UV light curing process. The scaffolds were fabricated under various temperature conditions, and it was confirmed that the stable fabrication was achieved when the temperature of the cooling jacket was 7 ± 2 °C and the cooling stages was 5 ± 2 °C. We investigated various physical properties (swelling ratio, water-uptake ability, Young’s modulus and degradation test) and cellular activities (initial attachment, morphological analysis, proliferation and osteogenic differentiation) on the prepared composite scaffolds (GS 0, GS 3 and GS 10). The composite scaffolds with silanated silica particles showed remarkable physical properties including improved tensile properties with increased Young’s modulus (12–23%) compared to the control group. Moreover, higher silica content not only led to a stable initial adhesion of the cells, but also excellent results in proliferation and differentiation of hMSCs. In particular, it was found that calcium deposition, the most basic bone differentiation index, elongated at a maximum of 185% compared to the control group. Based on these physical and biological results, it is believed that GelMA/silanated silica composite scaffolds have a potential to become a promising implantable material in the field of hard tissue regeneration.

## Funding

This research was supported by Priority Research Centers Program through the National Research Foundation of Korea (NRF) funded by the Ministry of Education (NRF-2018R1D1A1B07049434) and supported by the Technology development Program (S2839376) funded by the Ministry of SMEs and Startups (MSS, Korea) and also was supported by Priority Research Centers Program through the National Research Foundation of Korea (NRF) funded by the Ministry of Education (NRF-2020R1F1A1056503). 

*Conflict of interest statement*. None declared. 
